# The identification of sympatric cryptic free-living nematode species in the Antarctic intertidal

**DOI:** 10.1371/journal.pone.0186140

**Published:** 2017-10-05

**Authors:** Matthew R. Lee, Cristian B. Canales-Aguirre, Daniela Nuñez, Karla Pérez, Crisitan E. Hernández, Antonio Brante

**Affiliations:** 1 Centro i~mar, Universidad de Los Lagos, Puerto Montt, Chile; 2 Departamento de Ecología, Universidad Católica de la Santísima de Concepción, Concepción, Chile; 3 Laboratorio de Ecología Evolutiva and Filoinformática, Departamento de Zoología, Facultad de Ciencias Naturales y Oceanográficas, Universidad de Concepción, Concepción, Chile; 4 Centro de Investigación en Biodiversidad y Ambientes Sustentables (CIBAS), Universidad Católica de la Santísima de Concepción, Concepción, Chile; Laboratoire de Biologie du Développement de Villefranche-sur-Mer, FRANCE

## Abstract

The diversity of free-living nematodes in the beaches of two Antarctic islands, King George and Deception islands was investigated. We used morphological and molecular (LSU, and two fragments of SSU sequences) approaches to evaluate 236 nematodes. Specimens were assigned to at least genera using morphology and were assessed for the presence of cryptic speciation. The following genera were identified: *Halomonhystera*, *Litoditis*, *Enoploides*, *Chromadorita*, *Theristus*, *Oncholaimus*, *Viscosia*, *Gammanema*, *Bathylaimus*, *Choanolaimus*, and *Paracanthonchus*; along with specimens from the families Anticomidae and Linhomoeidae. Cryptic speciation was identified within the genera *Halomonhystera* and *Litoditis*. All of the cryptic species identified live sympatrically. The two cryptic species of *Halomonhystera* exhibited no significant morphological differences. However, *Litoditis* species 2 was significantly larger than *Litoditis* species 1. The utility of molecular data in confirming the identifications of some of the morphologically more challenging families of nematodes was demonstrated. In terms of which molecular sequences to use for the identification of free-living nematodes, the SSU sequences were more variable than the LSU sequences, and thus provided more resolution in the identification of cryptic speciation. Finally, despite the considerable amount of time and effort required to put together genetic and morphological data, the resulting advance in our understanding of diversity and ecology of free-living marine nematodes, makes that effort worthwhile.

## Introduction

The nematodes are one of the most abundant and diverse groups of metazoans on the planet [1]. They can be found in a wide variety of terrestrial, marine and freshwater habitats, both as free-living individuals and as parasites of both plants and animals [2]. In fact, an estimate of free-living nematode diversity by Appeltans et al. [3] of 50,000 species suggests that 86% of the existing species remain to be discovered, and that field surveys have sofar only described between 6% and 56% of nematode diversity. Recent molecular studies of free-living marine nematodes have revealed cryptic speciation within a number of genera [4, 5, 6, 7] suggesting that their diversity may be even higher than currently recognised. The ubiquity, abundance and economic importance of nematodes has led to them being widely used in genetic and phylogenetic studies [8, 9, 10]. The free-living nematodes are members of the meiofauna, and in most marine sediments they are typically both the most abundant and diverse component [2]. Nematodes occupy most trophic niches, from predators through omnivores and biofilm grazers to bacteriovores. There are also species that exhibit highly specialised trophic strategies such as absorbing dissolved organic matter directly across their body surface [11] or forming symbiotic relationships with chemosynthetic bacteria [12].

Identifying cryptic speciation in organisms with limited morphological variation is a major challenge for molecular ecologists. Mitochondrial or nuclear DNA sequences when used with morphological data collected prior to the extraction of DNA significantly improve the identification of cryptic and hidden biodiversity. DNA Barcoding as a concept was developed by Hebert et al. [13], they proposed the use of a single fragment of mitochondrial DNA (mtDNA) as a global specific identifier. This has been used in several taxonomic phyla (e.g. fish [14], polychaetes [15], crustaceans [16]), where the use of DNA has permitted the identification of several new species within a broad taxa, and highlighted hidden biodiversity [13]. Using DNA sequences to distinguish between species is particularly useful when dealing with taxa, such as nematodes, where morphological differences between species are often minute and difficult to discern, and where in many parts of the world traditional taxonomic information based on morphology is scarce.

Though nematodes are an important and abundant group in marine sediments they are often ignored due to their diversity, perceived complex taxonomy and the lack of the necessary taxonomic expertise within research groups to identify them. This group has also received less attention because their anatomy is simple, and inter-specific differences in many families are often difficult to discern using light microscopy. Several studies have used molecular techniques to identify nematode species from a variety of environments worldwide (e.g. estuaries [17], deep-sea [18], soil [19]), but few if any have studied intertidal Antarctic nematodes. The current information concerning the diversity of nematodes in Antarctic waters is primarily confined to the deep-sea [20, 21, 22], based on samples collected during the ANDEEP (Antarctic benthic deep-sea biodiversity) series of cruises. A search of the SCAR-MarBIN portal indicates that there are currently 385 species of nematodes described for the Antarctic region [23], though this is the result of a much lower sampling effort compared to other regions of the globe.

The difficulties in identifying nematodes using only their morphology has lead to the development of complementary molecular techniques [24, 25, 26], through which identification accuracy and consistency can be improved. The application of molecular methods to species delimitation has uncovered an overwhelming amount of unrecognized cryptic diversity or two or more species hidden under one species name [27]. Obtaining DNA from individual organisms that may have volumes as small as 30 nl (e.g. *Halomonhystera*) presents a challenge [28], however methodologies are being continually refined resulting in improved extraction efficiencies [29, 30]. Traditionally nematode molecular taxonomy has focused on mtDNA (e.g. COI) and nuclear rDNA. For the latter, the small subunit ribosomal (SSU or 18S), and the large subunit ribosomal (LSU) have been the most popular [31, 32, 33]. Bhadury et al. [4], for instance, used the 18S rRNA fragment as a barcode for identifying estuarine and marine nematodes, demonstrating the usefulness of this DNA fragment for taxonomic identification. They also suggested that this region would be useful for investigating nematodes where sufficient taxonomic expertise does not exist. In another example, Tchesunov et al. [34], used morphology and genetics to describe two new species of *Halomonhystera* from the marine environment. Although, new techniques using high throughput sequencing (i.e. Next Generation Sequencing, NGS) have helped to identify hidden biodiversity from environmental DNA (eDNA), from environments such as water and soil [35], the nematodes are typically poorly characterised from a taxonomic perspective [35]. However, when using NGS approaches there are typically no morphological records (e.g. morphological measurements, video vouchers, or digitized images) kept that can be associated with the sequences obtained [36]. Thus, where cryptic speciation is encountered a careful reexamination of the morphology to discover if there are any physical differences is not possible. Thus, an important first step in making these NGS data useful to ecologists is to take qualitative samples from the study site, make morphological observations and take photographs, make camera lucida drawings or create video vouchers of individual specimens before sequencing the DNA from those voucher specimens [37]. With this baseline information, combining molecular with morphological information, WIMP (What’s In My Pot) applications can be developed for use by non-specialists.

The research presented here is the first look at nematodes from the intertidal in Antarctica, specifically the south Shetland Islands. Each specimen was identified through traditional morphological techniques, morphometric data was collected and digital photos or video vouchers were created prior to molecular analyses. Sequences were developed for three DNA fragments (SSU1, SSU2 and LSU) and where cryptic speciation was suspected, the morphometric and field data were examined to see if the cryptic groups coincided with differences in morphology, location or sub-habitat.

## Methods

### Field collection

**Fig 1:**
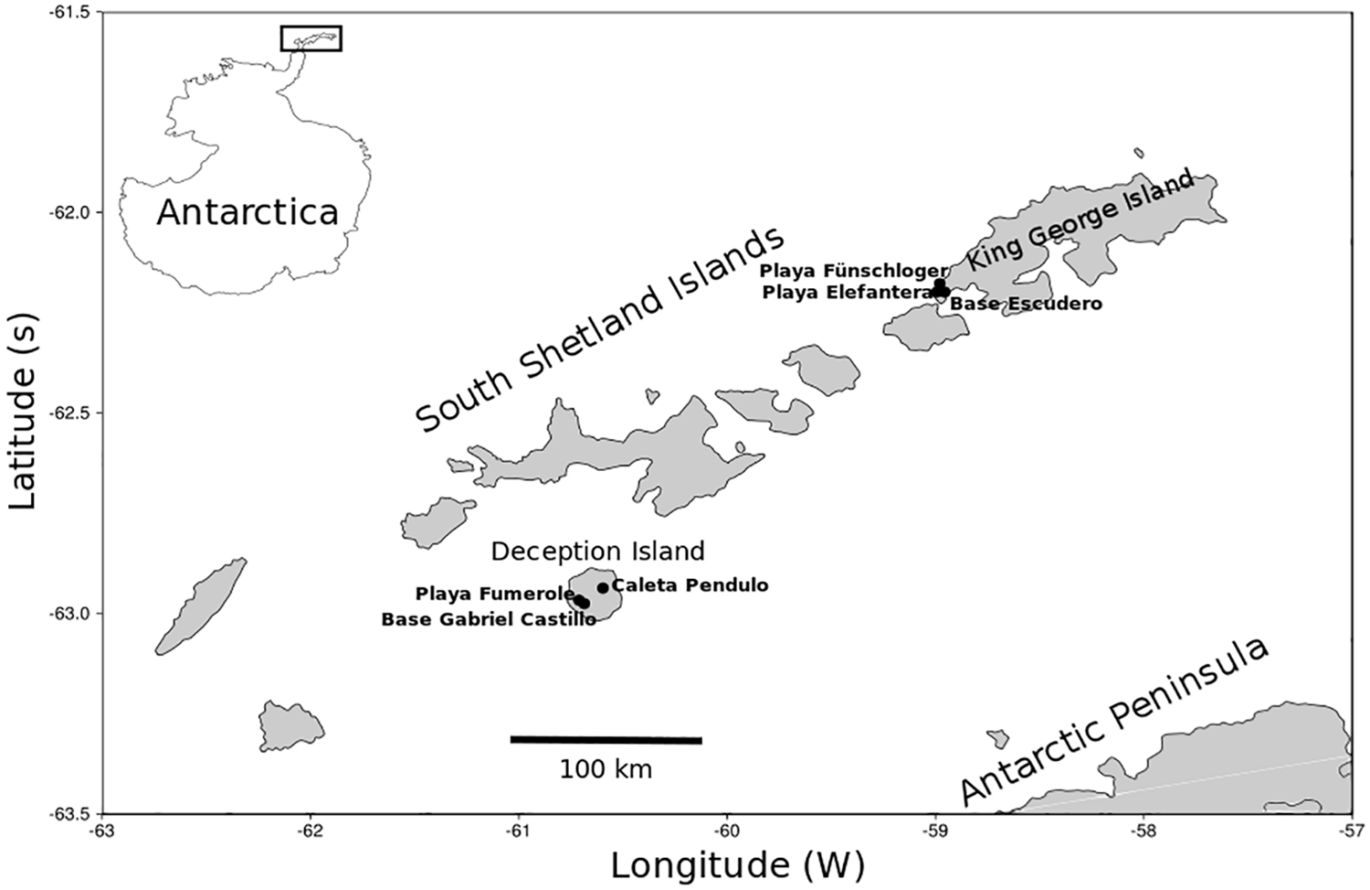
Map of the study sites on King George and Deception Islands, both located in the South Shetland Islands group off the Antarctic Peninsula.

Samples were collected during February 2012 at six sites: Playa Elefantera (62.19842 °S, 58.99414 °W), Playa Fünschloger (62.17722 °S, 58.97994 °W) and Base Escudero (62.19850 °S, 58.95583 °W) all on the Fildes Peninsula of King George Island; and Base Gabriel Castillo (62.97528 °S, 60.68957 °W), Playa Fumarole (62.96689 °S, 60.71083 °W) and Caleta Pendulo (62.93664 °S, 60.59647 °W) all within the flooded caldera of Deception Island (Fig 1). Qualitative samples were collected in a number of intertidal microhabitats at each site, including surface sediment from the high, mid and low shore; from the depth of the water table in the mid-shore (variable depth depending on the nature of the site), and macroalgae growing on adjacent rocky shores. The qualitative samples were collected by taking small quantities of sediment (or algae) from random points within a defined microhabitat using a small shovel up to a volume of approximately 500 ml. Sampling permissions for the study areas were granted by the Chilean Antarctic Institute INACh (http://www.inach.cl/inach/), in compliance with the Protocol on Environmental Protection in the framework of The Antarctic Treaty, and related agreements or the Antarctic Treaty System, ATS (http://www.ats.aq/index_e.htm).

### Fauna extraction

The meiofauna present in the sediment samples were extracted in the field before fixation. The extraction of the fauna was in two steps both using decantation [38]. First, fauna were extracted using 45 μm filtered seawater over a 45 μm mesh, then the sediment sample was flooded with isotonic magnesium chloride (MgCl_2_, 75g L^-1^), left for 15 minutes for anaesthetisation of the fauna to take place and then decanted over a 45 μm mesh. The extracted fauna were then fixed in DESS, a solution of dimethyl sulphoxide, disodium EDTA, in a saturated NaCl solution [39]. This solution has the advantage of preserving the fauna well enough to be identified using morphology but also without damaging the DNA.

### Species identification and morphology

In the laboratory individual nematodes were picked out of the samples and placed on temporary mounts. Individuals were then identified to the lowest taxonomic level possible, typically genera (Table 1). Individuals were also drawn, photographed and/or video vouchered. Morphometric measurements were also made: length, maximum width, tail length, oesophageal length and position of the vulva (in females), and De Man ratios a, b and c, the ratios of total body length to maximum diameter, oesophagus length and tail length respectively, were calculated [40]. Individuals were then recovered from the mounts and stored individually in 0.2 ml PCR tubes in DESS and sent for DNA extraction.

### DNA extraction and PCR amplifications

The total DNA was isolated from the entire individual using the Chelex 5% protocol (BIORAD^™^). The LSU and two SSU fragments from nuclear ribosomal DNA coding regions (~400 bp) were used as diagnostic sequences, which have been successfully used in previous nematode studies [41]. The LSU was amplified using the primers A-D3A / B-D3B [42] and the SSU using the primers MN18F / 22R [4, 32] (referred to hereafter as SSU1) and A-NF1 / B-18Sr2b [43] (referred to hereafter as SSU2). All these pairs have previously been used for the molecular identification of nematodes [32, 41, 43]. The SSU1 primer pair correspond to the nucleotide positions 54–74 and 377–395 for MN18F and 22R respectively. The SSU2 primer pair correspond to the nucleotide positions 1210–1253 and 1575–1614 for A-NF1 and B-18Sr2b respectively. All these occur in the sequence of 18s rDNA from *Geomonhystera disjuncta* ([9]; GenBank accession number AJ966485).

The amplification of SSU1 was conducted in a final volume of 50 μl, and for SSU2 and LSU in a final volume of 25 μl. The PCR conditions were: a) for SSU1, 5 μl of template DNA, 2.5 μl of Taq’s polymerase buffer with MgCl_2_, 0.5 μl of dNTPs, 0.5 μl of each primer, and 0.165 μl of TopTaq DNA polymerase (Qiagen^®^); b) for SSU2 and LSU, 1 μl of template DNA, 2.5 μl of Taq’s polymerase buffer with MgCl_2_, 0.5 μl of dNTPs, 0.5 μl of each primer for SSU2 and 1 μl for LSU, and 0.165 μl of Taq DNA polymerase (Invitrogen^®^). The PCR amplification of SSU1 fragments was conducted with the following parameters: an initial denaturation at 95°C for 300 seconds, followed by 37 cycles of denaturation at 95°C for 30 seconds, annealing at 56°C for 60 seconds, extension at 72°C for 90 seconds, and a final elongation step at 72°C for 300 seconds. For SSU2 amplification of fragments was conducted with the following parameters: an initial denaturation at 94°C for 600 seconds, followed by 35 cycles of denaturation at 94°C for 60 seconds, annealing at 58°C for 30 seconds, extension at 72°C for 60 seconds. For LSU fragments amplification was conducted with the following parameters: an initial denaturation at 95°C for 300 seconds, followed by 35 cycles of denaturation at 95°C for 60 seconds, annealing at 55°C for 60 seconds, extension at 72°C for 120 seconds, and a final elongation step at 72°C for 600 seconds. All PCR amplifications were performed in a Bioer thermal cycler. The PCR products were visualized in 2% agarose gel stained with ethidium bromide (1μl). The samples were sequenced at the Macrogen^®^ Company (Korea) in an automated DNA sequencer (Model 3730xl; Applied Biosystems), and sequences were deposited in GenBank under following accession numbers LSU: KY792262—KY792388, SSU: KY792389—KY792534, and 18S: KY792087—KY792261.

### BLAST-match searching at NCBI

Each LSU, SSU1, and SSU2 sequence obtained was checked using the Basic Local Alignment Search Tool (BLASTn, [44]) at NCBI. To optimize the BLAST searching, each BLAST examination was restricted to the phylum Nematoda (taxid:6231). A low expected value (e-value = 1) was used in order to be more stringent, and avoid increasing the number of BLAST hits by chance. The e-value is the number of the BLAST hits (alignment) that might be expected to be found by chance, with the observed score or higher. Thus, the three most similar sequences matched with the NCBI database with the highest percentage in coverage and lowest e-value were recorded. This information was then checked against the morphological identifications made for each specimen.

### Molecular taxonomy by phylogenetic analyses

All chromatograms were visually checked for base-calling errors, and poor quality reads were removed using clip parameters set to “maximize region with error rate below 0.01” using CodonCode Aligner 2.0.1 (CodonCode Corporation, Dedham, MA, USA). Initial alignments were performed with the CodonCode Aligner and final alignments were adjusted by eye. The alignment by eye was carried out in windows of 120 bp, within which likely errors can be detected visually. Only a few nucleotides were adjusted manually post-alignment. The interspecific phylogenetic analysis was conducted using a Bayesian Markov Chain Monte Carlo approach (BMCMC), which incorporates the uncertainty in the reconstruction of the phylogenetic tree. With the BMCMC approach, a general likelihood-based mixture model of gene-sequence evolution was applied as described by Pagel and Meade [45, 46]. The latter was used because the molecular marker used in this study included fragments of LSU, SSU1, and SSU2 genes that could have several patterns and rates of nucleotide substitution. This mixture model, based on the general time-reversible (GTR) model [47], accommodates cases in which different sites in the alignment evolved in qualitatively distinct ways, but does not require prior knowledge of these patterns or partitioning of the data. A mixture model, implemented within a BMCMC framework, was used to estimate the posterior probability of the intraspecific phylogenetic trees and to include this information in further comparative method analyses. The Reversible-Jump Markov Chain Monte Carlo procedure [48, 49] was used with the objective of integrating the results of all patterns, and a mixture model that summarised the sequence evolution, was produced using BayesPhylogenies v1.1 software (http://www.evolution.rdg.ac.uk/BayesPhy.html). This approach enabled the exploration of a variety of possible models and parameters, converging towards the model that best fits the data in the posterior tree sample [49]. Independent BMCMC analyses were run using 10,000,000 generations of phylogenetic trees, sampling every 10,000th tree to assure that successive samples were independent. From this sample of trees, the first 5% were removed to avoid including trees sampled before the convergence of the Markov Chain. A final sample of 950 trees was obtained, and these trees were used to obtain the phylogenetic consensus tree, and their posterior probabilities.

### Molecular Operational Taxonomic Units (MOTU)

Following the method described by Floyd et al. [32] and Blaxter [50], different Molecular Operational Taxonomic Units (MOTU) were identified in the data set. Sequences were assembled using an end to end alignment algorithm, and with a minimum percentage of identity (i.e. 99%) and then MOTU were identified for each gene and the concatenated information. In addition, species delimitation analysis was performed with the Poisson Tree Processes (PTP) including a Bayesian implementation of the model [51], using the phylogenetic consensus tree based on the concatenated genetic datasets. The estimations were made using the bPTP web server (http://species.h-its.org/ptp/), with 500,000 MCMC generations, thinning set to 100, burning at 10%, Seed = 123 and with the outgroup removed for the Bayesian search. The probability of each node representing a species node was calculated in two ways: 1. The maximum likelihood solution; and 2. the highest Bayesian supported solution, taking into account the frequency of the nodes across the samples.

### Analysis of the morphology and habitat characteristics of the identified cryptic groups

The constructed trees for LSU, SSU1, SSU2 and concatenated sequences were used to identify the presence of potential cryptic species within each genera. The morphometric data of each of these potential cryptic species was then analysed to determine if there were any significant differences between them. This was done using one-way ANOVA for each of the measures taken: length, maximum width, oesophageal length, tail length and the De Man ratios a, b and c. All statistical analyses were conducted using R [52]. Data were tested for normality and homogeneity of variances prior to analysis and where necessary data were transformed using Box-Cox power transformations.

## Results

### Specimens

236 specimens were identified belonging to 12 families and sent for sequencing (Table 1). Not all specimens yielded usable sequences (Table 1). Where cryptic species were identified the morphological data collected was used to determine whether there were any significant body-size differences between these cryptic species (see below; S4 Table). The following genera were identified: *Halomonhystera* (= *Geomonhystera*, = *Monhystera*, [53]); *Litoditis* (= *Pellioditis*, = *Rhabditis*, [54]); *Enoploides*; *Chromadorita*; *Theristus*, *Oncholaimus*, *Viscosia*, *Gammanema*; *Bathylaimus*; *Choanolaimus*; and *Paracanthonchus*; along with specimens from the families Anticomidae and Linhomoeidae.

**Table 1:** Number of specimens sequenced per species for each site and the proportion of specimens that produced a usable sequence for the genes LSU, SSU1 and SSU2, and the proportion of specimens that produced no usable sequences. The final three rows of the table present the number of specimens and the average proportions for the two islands sampled and for all the sites. Samples were collected during February 2012 at six sites: Playa Elefantera (ELE), Playa Fünschloger (FUN) and Base Escudero (ESC) all on the Fildes Peninsula of King George Island; and Base Gabriel Castillo (CAS), Playa Fumarole (FUM) and Caleta Pendulo (PEN), on Deception Island.

Site	Specimens	LSU (%)	SSU1 (%)	SSU2 (%)	No Sequence (%)
**ESC**	40	45.0	90.0	77.5	2.5
**ELE**	60	60.0	73.3	90.0	3.3
**FUN**	120	67.5	84.2	55.8	2.5
**CAS**	9	33.3	44.4	11.1	22.2
**FUM**	5	20.0	20.0	40.0	60.0
**PEN**	2	50.0	0.0	50.0	50.0
**King George**	220	57.5	82.5	74.4	2.8
**Deception**	16	34.4	21.5	33.7	44.1
**All Sites**	236	46.0	52.0	54.1	23.4

### BLAST-match searching at NCBI and MOTU

From 236 individuals sequenced from six sampling locations, a total of 488 sequences (LSU = 133, SSU1 = 196, and SSU2 = 159) were obtained. These sequences were used for further analyses. Almost all sequences obtained in this study matched with the phylum Nematoda, the exceptions were those that matched with species of Fungi (Cryptococcus, Tilletiopsis, Lecanicillium, and Brachyalara; S1 Table). These sequences that did not match with the Nematoda were removed from further analyses. For LSU eight families were represented (Rhabditidae, Thoracostomopsidae, Monhysteridae, Oncholaimidae, Cyatholaimidae, and Xyalidae), for SSU1 9 families (Chromadoridae, Cyatholaimidae, Monhysteridae, Oncholaimidae, Rhabditidae, Selachinematidae, Thoracostomopsidae, Tripyloididae, and Xyalidae), and for SSU2 12 families (Monhysteridae, Anticomidae, Rhabditidae, Choanolaimidae, Oncholaimidae, Cyatholaimidae, Leptolaimidae, Linhomoeidae, Chromadoridae, Thoracostomopsidae, Tripyloididae, and Xyalidae) (S2 Table). The e-values were extremely low in almost all the sequences and fragments used in this analysis. These ranged from 7e^-61^ and 4e^-153^ for LSU, 1e^-75^ and 4e^-109^ for SSU1, and 9e^-95^ and 5e^-177^ for SSU2 for the first match in BLAST hits (alignment). These outcomes confirm that the sequences used for further analyses (see next section *Molecular taxonomy by phylogenetic analyses*) were not chosen by chance, but by homology (S1 Table). Taking 99% similarity as the cut-off point the SSU1 and SSU2, along with the concatenated data, produced the most MOTU (19), while the less variable LSU produced fewer (14) (S3 Table).

### Molecular taxonomy by phylogenetic analyses

**Fig 2:**
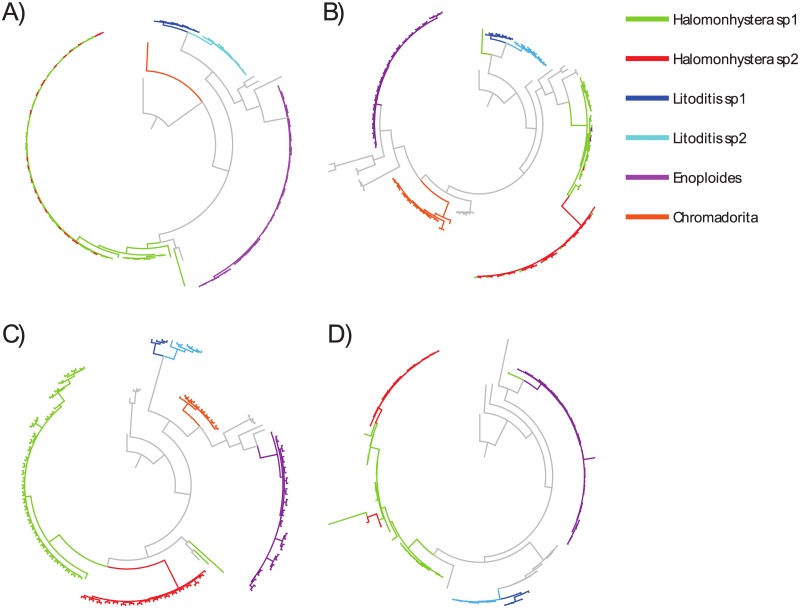
Bayesian phylogenetic tree constructed for A) LSU, B) SSU2, C) SSU1, and D) combine sequences. Colours are related to the morphological identifications in S1 Table.

**Fig 3:**
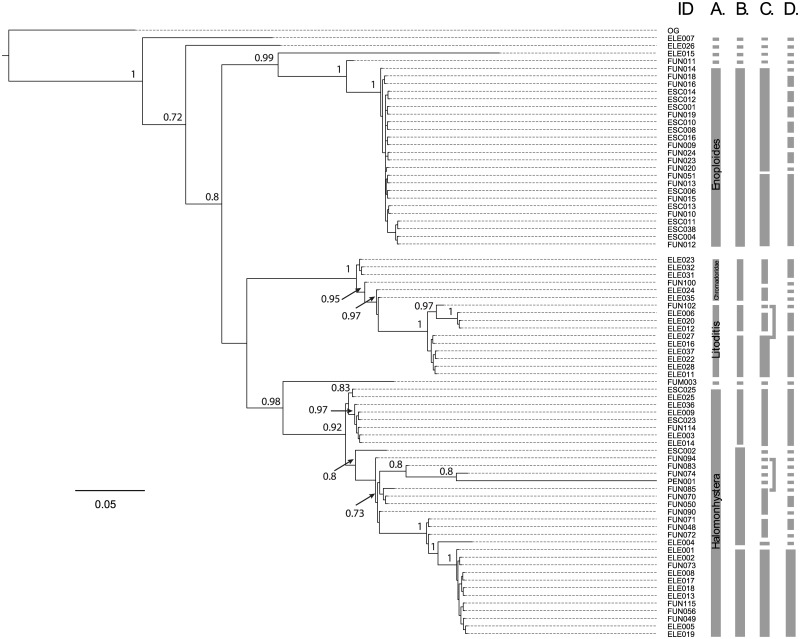
Bayesian consensus phylogenetic tree of nematodes collected in Antarctica inferred by LSU, SSU1, and SSU2 gene fragments. The grey columns indicate the species identified by the different approaches. ID indicates the specimen identification number. A. indicates the species identified only by morphology (see S1 Table); B. indicates the putative species identified by high support clusters obtained with the SSU1 phylogenetic tree for the more abundant morphological species (e.g. *Halomonystera* and *Litoditis*, S3 Fig); C. indicates the putative species identified using the Molecular Operational Taxonomic Units approach, the cut-off value was a similarity of 99%; D. indicates the putative species identified using the Bayesian implementation of the Poisson tree processes (bPTP) model for species delimitation based on the molecular phylogenetic consensus tree.

Phylogenetic reconstructions showed several clades supported by a posterior probability larger than 0.5 (Fig 2). The supported clades for all one-gene reconstructions exhibited no geographical pattern, therefore individuals from different locations were aggregated. The concatenated tree presented the highest values of posterior probabilities for almost all clades, supporting the use of more than one locus to improve the phylogenetic reconstructions. The main clades in the LSU phylogenetic tree (Fig 2A, S1 Fig) included *Halomonhystera* (pp = 0.67), *Enoploides* (pp = 0.88), and *Litoditis* (pp = 0.96). For SSU1 the main clades of the phylogenetic tree supported by posterior probability included *Enoploides* (pp = 0.65), and *Litoditis* (pp = 0.99) (Fig 2C, S2 Fig). For SSU2 the phylogenetic tree main clades included *Halomonhystera* (pp = 0.95), *Enoploides* (pp = <0.5), *Litoditis* (pp = 0.71), and Chromadoridae (pp = 0.99) (Fig 2B, S3 Fig). The concatenated tree showed the same main clades of *Halomonhystera* (pp = 0.92), *Enoploides* (pp = 1.0), and *Litoditis* (pp = 1.0) (Fig 2D). All phylogenetic trees built resolved two well-differentiated clades for the genera *Halomonhystera* and the *Litoditis* (Fig 2). In addition, in all phylogenetic trees other less representative clusters included Oncholaimidae, Cyatholaimidae, Tripyloididae, Selachinematidae, Xylidae (Fig 2). Both, the maximum likelihood and the Bayesian solution of the bPTP analyses suggested that there were between 32 and 39 species (Acceptance rate: 0.120988, Merge: 249699, and Split: 250301), resulting in a mean value of 35 putative species (Fig 3).

### Analysis of the morphology and habitat characteristics of the identified cryptic groups

*Halomonhystera* sp.: The SSU1 tree identified two potential cryptic species within the *Halomonhystera*, *Halomonhystera* sp.1 and *Halomonhystera* sp.2, these cryptic species were supported by the SSU2 tree though the separation was less clear. The LSU tree also identifies two groups, though only one shows a high degree of consistency, in terms of the assignment of specimens, with *Halomonhystera* sp.1(SSU1). Statistical analysis of the morphometric data for the two potential cryptic species indicated by SSU1 reveals no significant differences in morphology between them (Length: d.f. = 1, F = 1.226, p = 0.271; Width: d.f. = 1, F = 0.004, p = 0.947; Oesophageal length: d.f. = 1, F = 2.384, p = 0.126; Tail length: d.f. = 1, F = 0.237, p = 0.627; De Man ratio a: d.f. = 1, F = 1.214, p = 0.273; De Man ratio b: d.f. = 1, F = 0.047, p = 0.829; De Man ratio c: d.f. = 1, F = 0.373, p = 0543). Neither of the two cryptic species of *Halomonhystera* identified were associated with a specific site nor with a specific microhabitat within the sites.

**Fig 4:**
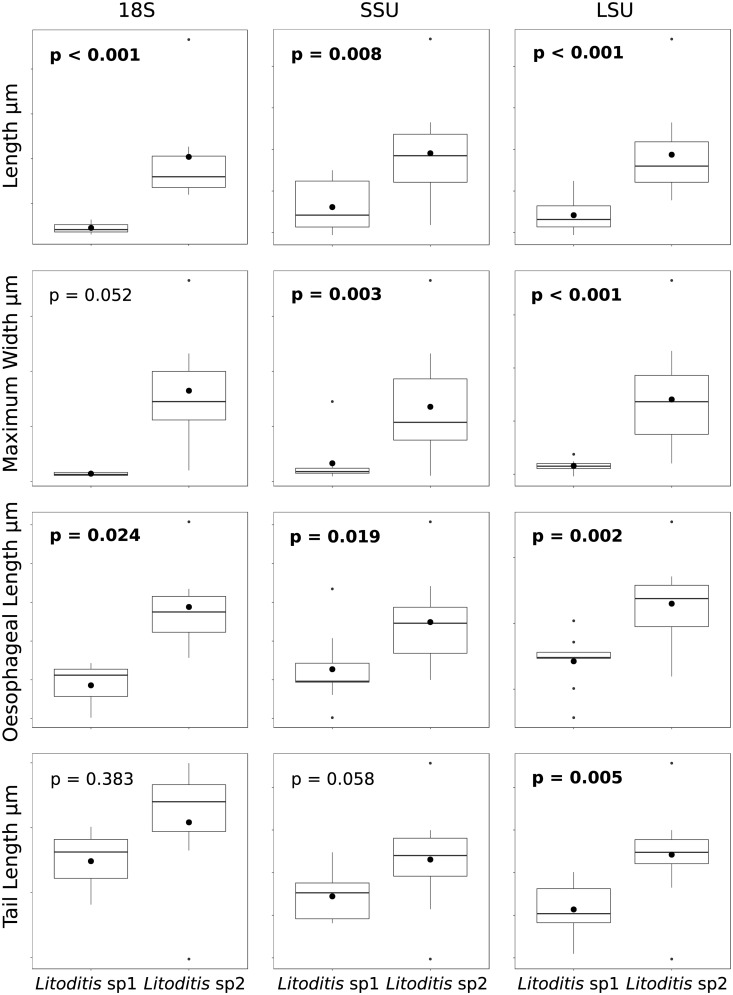
A comparison of the morphometric measurements for the two cryptic species of *Litoditis* identified by the molecular analyses. The p values correspond to the results of one-way ANOVA.

*Litoditis* sp.: Each of the trees (SSU1, SSU2 and LSU) identified two potential cryptic species within the *Litoditis*, *Litoditis* sp.1 and *Litoditis* sp.2. Specimens were consistently assigned across trees to the same cryptic species. Statistical analysis of the morphometric data indicated that *Litoditis* sp.2 (SSU1 n = 4, SSU2 n = 9, LSU n = 9) is larger than *Litoditis* sp.1 (SSU1 n = 7, SSU2 n = 12, LSU n = 11) (Fig 4). Body length was significantly longer in *Litoditis* sp.2 than in *Litoditis* sp.1 for all genes (SSU1, d.f. = 1, F = 40.990, p < 0.001; SSU2, d.f. = 1, F = 8.940, p = 0.008; LSU, d.f. = 1, F = 26.930, p < 0.001). Maximum body width was significantly wider in *Litoditis* sp.2 than in *Litoditis* sp.1 for the genes of SSU2 and LSU, but not for SSU1 (SSU1, d.f. = 1, F = 5.231, p = 0.052; SSU2, d.f. = 1, F = 12.000, p = 0.003; LSU, d.f. = 1, F = 34.560, p < 0.001). Oesophageal length was significantly longer in *Litoditis* sp.2 than in *Litoditis* sp.1 for all genes (SSU1, d.f. = 1, F = 7.771, p = 0.024; SSU2, d.f. = 1, F = 6.632, p = 0.020; LSU, d.f. = 1, F = 13.530, p = 0.002). Finally tail length was significantly longer in *Litoditis* sp.2 than in *Litoditis* sp.1 only in the case of the LSU gene (SSU1, d.f. = 1, F = 0.851, p = 0.383; SSU2, d.f. = 1, F = 4.101, p = 0.058; LSU, d.f. = 1, F = 13.530, p = 0.005). The proportional dimensions of *Litoditis* sp.1 and *Litoditis* sp.2, as indicated by the De Man ratios a, b and c, were not significantly different for any of the sequences. The combined inference of these results is that *Litoditis* sp.2 is significantly larger than *Litoditis* sp.1 but the body proportions are the same. Neither of the two cryptic species of *Litoditis* identified were associated with a specific site nor with a specific microhabitat within the sites. Furthermore, both males and females were assigned to each of the cryptic species, therefore the size differences observed were not due to sexual dimorphism.

## Discussion

The phylogenetic reconstruction using the sequences generated in this study indicate the presence of cryptic species within the nematode assemblages of the Antarctic intertidal beaches studied (Fig 3), specifically within the genera *Halomonhystera* and *Litoditis* (Fig 2). All of the cryptic species identified live sympatrically, that is to say that no single cryptic species was confined to a particular microhabitat or site, and that all microhabitats or sites contained at least two or more cryptic species. That said, the specimens of *Halomonhystera* from Deception Island were in the main placed apart on the trees from the specimens from King George Island. However, given the paucity of specimens from Deception Island far more data is required before this can be interpreted as cryptic speciation. In the case of the two cryptic species of *Halomonhystera* identified using SSU1 no differences in morphology were detected, which might be indicative of incomplete lineage sorting [55], resulting in a lack of reciprocal monophyly (Fig 2). On the other hand, of the two cryptic species of *Litoditis*, cryptic species 2 was consistently and significantly larger than cryptic species 1 (Fig 4). The differences in the morphology of the two *Litoditis* species were not discernible during the initial observations, only becoming evident after the statistical analysis of the morphometric data. The bPTP analyses identified a number of putative species within the main genera identified (*Halomonhystera*, *Litoditis*, *Enoploides* and *Chromadorita*) with nine distinct clades identified within *Enoploides*. Though as most of these clades comprise of only one or two specimens statistical analyses of potential morphological differences were not possible.

Both *Halomonhystera* and *Litoditis* feed on bacteria associated with decaying macroalgae [56, 57] and this explains why far more individuals of these two genera were found at Elefantera and Fünschloger compared to the other sites sampled (Table 1); as at both these sites, on the exposed coast of the Fildes peninsula on King George Island, large quantities of macroalgae are deposited on the beaches. The other sites sampled were more protected and not subject to the deposition of large quantities of macroalgae. In addition Deception Island is an active volcano [58] and the sampling sites were located within the flooded caldera where water temperatures can reach 30°C or more, an extreme environment for Antarctic species that have evolved for temperatures closer to zero.

Given that the identified cryptic species live sympatrically the question is: what is driving their speciation? It has been suggested that cryptic species of both *Halomonhystera* and *Litoditis* may coexist due to differences in their associated microbiome (the range of bacteria consumed is quantitatively different between species) [56]. Currently not much is known about the Antarctic microbiome where the samples were collected, however there is evidence of differences in bacterial diversity across the Fildes Peninsula [59]. Cryptic species may also coexist due to density dependant differences in dispersal [60]. Another possibility is niche differentiation due to differences in thermal tolerance [61], the Antarctic intertidal is an extremely stressful thermal environment, particularly in the summer where temperatures can range from -2.5°C to +22°C. Thus the high frequency of cryptic speciation observed in these genera at different locations around the world may be due to their high degree of niche specificity combined with their relatively short generation times (*Litoditis marina*, 5 days, [62]; *Halomonhystera disjuncta*, 6 days, [63]) and lack of a planktonic dispersal phase. Though it should be taken into consideration that generation times vary with temperature, the lower the temperature the longer the generation time [54, 64]. Thus in the Antarctic there may be considerable seasonal variation in generation times, with rapid population increases in summer with a much smaller over wintering population with much longer generation times, or a winter population in diapause [65, 66]. These factors in combination may result in rapid adaptation to changing environmental conditions and food availability, ultimately leading to speciation. Tchesunov et al. [34] found two new species of *Halomonhystera* living in distinct habitats: 1) *H*. *hermesi* sp. n. in bacterial mats in deep water sites in the Barents Sea, and 2) *H*. *socialis*, occurring on mass on detached kelp accumulated in the upper sublittoral. These species were identified as a result of differences in their DNA sequences, and subsequently supported by differences in morphology, again highlighting the importance of collecting morphometric data prior to the sequencing of specimens.

Nematodes collected on Deception Island included the genera *Halomonhystera* and *Theristus* (from the sister family Xylidae). These specimens exhibited longer branch lengths in the phylogenetic tree than specimens of *Halomonhystera* collected on King George Island. This suggests that the mutation rate which is related to branch length, varies considerably in antarctic nematodes (see also: [67, 68]). High substitution rates have been associated with parasitic lifestyles and/or short generation times [68]. However, the two genera mentioned differ in generation times. Short generation times are typical for free-living nematodes, especially “r-selected” species such as those of the genera *Halomonhystera* and *Litoditis*, from days [54, 62, 63] to weeks [69]. In the case of *Theristus* generation times may be longer, from months [69] to a year [70]. It should however be pointed out that the majority of estimates of generation times in free-living nematodes come from laboratory culture experiments that may not be representative of what occurs in the natural, more variable, environment; and as mentioned above generation times can vary considerably with temperature. Thus the high substitution rates implied in nematodes from Deception Island may be a result of not simply short generation times but the selective pressures of the extreme habitat in which the nematodes are living, a beach in the flooded caldera of an active volcano in Antarctica.

The genera *Enoploides* are assumed to be predators [71, 72] and the sequence data confirms this where a number of specimens identified as *Enoploides* produced sequences placed within the *Halomonhystera* or *Litoditis* clades. Misidentification of the *Enoploides* specimens is unlikely as they are morphologically quite distinct from the other two genera. Thus these “unexpected” sequences should be interpreted as evidence that *Enoploides* is a predator of both *Halomonhystera* and *Litoditis* which is logical given that those two genera were the most abundant nematodes present in the sediment. This confirmation of the trophic guild of *Enoploides* and the identification of the prey species is valuable ecological information. Nematodes are generally assigned to trophic guilds on the basis of their buccal morphology, with certain buccal morphologies associated with certain food categories [73, 74]. However, this entire categorisation is based on the whole on assumptions, as direct observations of nematodes are absent for the vast majority of species, and recent experiments using stable isotopes, for example, have revealed the trophic strategies of nematodes may be more complex than previously thought [75]. Thus, molecular techniques could prove an important tool in determining, or confirming, the trophic guilds of individual species. Another example of “unexpected” sequences arising from specimens identified as nematodes are the fungal sequences produced. All organisms have a microflora associated with their epidermis and gut, and in some cases living symbiotically in their tissues (e.g. Microsporidia, [76]). These “unexpected” sequences highlight the issues that could arise from relying on only molecular data and should be taken into consideration when evaluating the results of metagenomic and eDNA analyses. What is being sequenced the organism or its lunch? On the other hand, as described by [77, 78] relying on morphological identifications alone can in some cases, due to morphological variation and plasticity, lead to an over estimation of diversity.

A number of specimens were identified morphologically as a species of the genera *Chromadorita*, family Chromadoridae, and these sequences produced a clear well defined group (Fig 2B and 2C). However, comparing with the sequences available in genbank the closest matches were with *Neochromadora* (SSU1) and *Dichromadora* (SSU2), no match was found for the LSU sequences which seems to be a less variable gene in the case of nematodes. The species found in Antarctica was lacking the pronounced lateral differentiation on the cuticle, which is characteristic of both *Neochromadora* and *Dichromadora*, but not present in *Chromadorita*. This may be illustrative of a general issue of using sequences for identification purposes as there are currently insufficient sequences in genbank to provide accurate identifications [79]. Thus there is a pressing need for nematodes of a wide diversity of families to be sequenced from a wide diversity of habitats and geographic locations, in order to make tools such as metagenomics useful for generating species lists in environmental and ecological studies. This is especially important for morphologically complex and diverse families like Chromadoridae which are notoriously difficult to identify to species without considerable experience (e.g. [80]).

Sofar investigations of cryptic diversity within free-living marine nematodes have been restricted to a relatively small number of families/genera. Cryptic speciation within the genera *Halomonhystera* and *Litoditis* has been described in a number of papers and from a number of different habitats from the intertidal [17, 81] and estuarine habitats [56, 81] down into the deep sea around seeps [6, 63, 82]. These genera also have a global distribution, from the Arctic [57] down to the Antarctic [83]. Other families where cryptic speciation has been investigated include: Linhomoeidae (*Terschellingia longicaudata*, [84]); Leptosomatidae *(Thoracostoma trachygaster*, [5]); Thoracostomopsidae [77]; and Desmodoridae [85, 86]. Some of these species are described as having a cosmopolitan distribution (e.g *Halomonhystera disjuncta*, *Litoditis marina*, *Terschellingia longicaudata*) but this is based on morphological descriptions, and the application of molecular methods that can identify cryptic diversity can cast doubt on the status of many cosmopolitan species [27]. Thus the next step should be international collaborations to see if the different cryptic species have a similar cosmopolitan distribution or whether they are more restricted geographically. Furthermore, given the extremely high diversity and dominance of nematodes in benthic marine environments a much wider range of families/genera need to be assessed for the presence of cryptic speciation, in order to give a much better estimate of the incidence of cryptic speciation and how this might affect global diversity estimates. Setting aside how cryptic speciation can affect biodiversity estimates, it may also highlight population genetic structure, as was found by [5] for *Thoracostoma trachygaster* using reverse taxonomy and morphology.

There is no doubt that direct sequencing of environmental samples using NGS has contributed to the knowledge of the total biodiversity that is encompassed by the meiofauna [41, 87, 88]. However, NGS technology is still developing and current results need to be treated with caution due to the potential for under- or overestimation of species richness due to variable sequencing efficiences [88]. To that end, tradicional DNA sequences (e.g. SSU, LSU, COI) obtained by Sanger methodology are highly valuable and still widely used for nematode taxa (e.g. [5, 10, 89, 90]). Both methods, NGS and Sanger, have advantages and disadvantages [91], nonetheless to move forward in disentangling of the hidden diversity of nematode assemblages, matching DNA sequences with morphology and additional metadata (e.g. gender, habitat, environmental variables, etc), is a vital step in enhancing the utility of molecular data [41, 92]. For example, the reverse taxonomy proposed by Markmann and Tautz [93] for meiobenthic organisms, is a good way of linking DNA to a putative species or recent species delimitation methods based on Bayesian approaches (e.g. [94]).

Finally, despite the considerable amount of time and effort required to put together genetic and morphological data, the resulting advance in the understanding of the diversity and ecology of free-living marine nematodes, makes that effort worthwhile. In this study the first information on the diversity of free-living nematodes in the intertidal beaches of the South Shetland Islands in Antarctica has been provided. Cryptic species were identified within three genera, *Halomonhystera*, *Litoditis* and *Enoploides*. In terms of which molecular sequences to use for the identification of free-living nematodes, both fragments of SSU were more variable than the LSU sequences, and thus provided more resolution in the identification of cryptic speciation. And finally, the study has also demonstrated the utility of molecular data in confirming the identifications of some of the morphologically more challenging families of nematodes.

## Supporting information

S1 Table List of all identification results using BLAST search of GenBank. (XLSX)

S2 Table Summary of families identified. (XLSX)

S3 Table Molecular Operational Taxonomic Units (MOTU). (XLSX)

S4 Table Morphological data. (XLSX)

S1 Fig LSU Bayesian tree. (PDF)

S2 Fig SSU1 Bayesian tree. (PDF)

S3 Fig SSU2 Bayesian tree. (PDF)
