# Supplementary figures and images for: The identification of sympatric cryptic free-living nematode species in the Antarctic intertidal
Source: PLoS One. 2017 Oct 5;12(10):e0186140. doi: 10.1371/journal.pone.0186140 (PMC5629031; doi:10.1371/journal.pone.0186140)

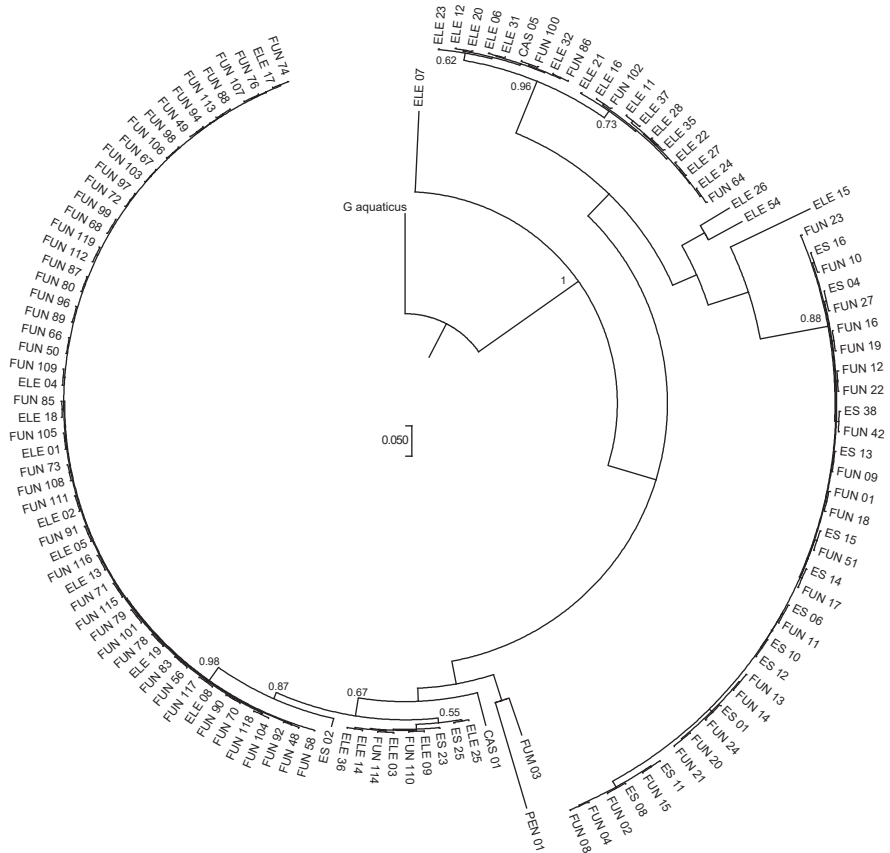

Supplement: S1 Fig — (PDF) [file pone.0186140.s005.pdf]

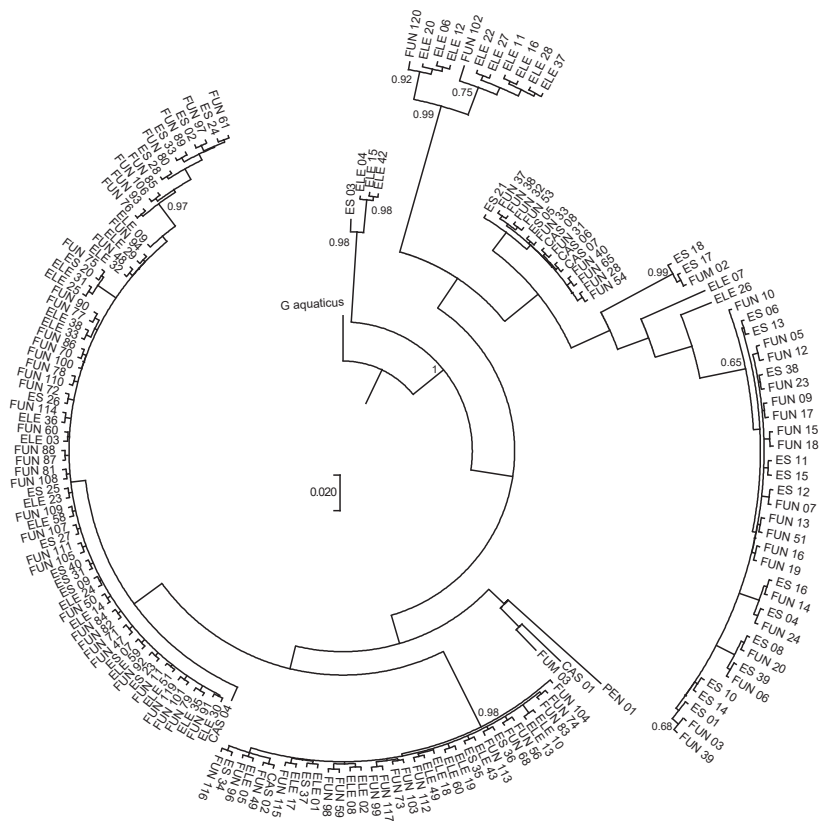

Supplement: S2 Fig — (PDF) [file pone.0186140.s006.pdf]

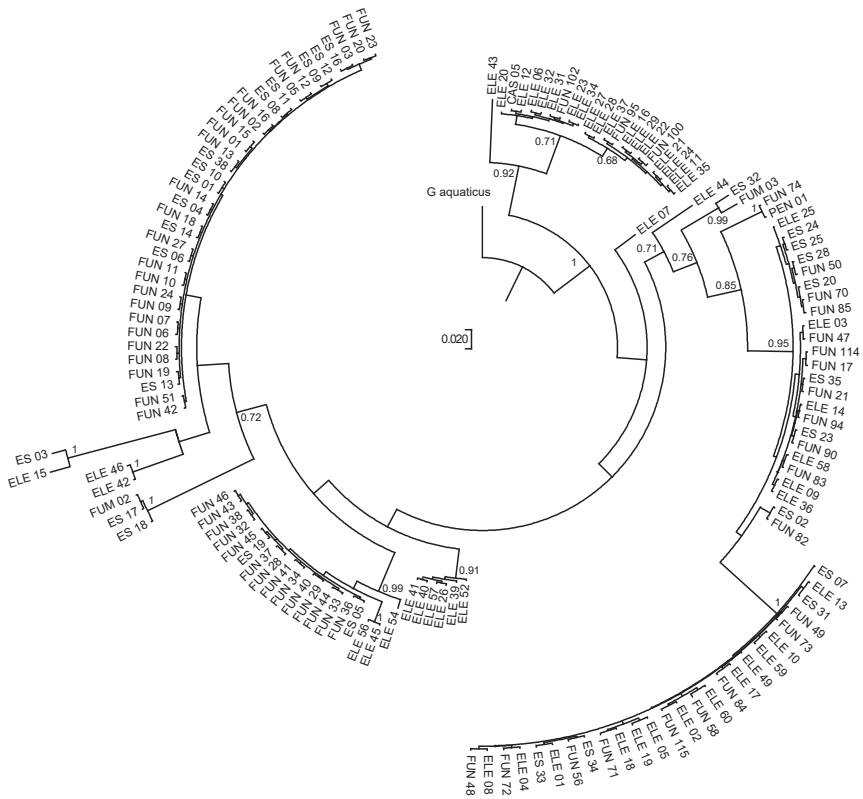

Supplement: S3 Fig — (PDF) [file pone.0186140.s007.pdf]
